# Pooled Shotgun Metagenomics Reveals Cloacal Microbiota Composition and Resistome Patterns in Chickens from Kazakhstan

**DOI:** 10.3390/microorganisms14081689

**Published:** 2026-08-01

**Authors:** Ilya Korotetskiy, Sergey Shilov, Tatyana Kuznetsova, Natalya Zubenko, Lyudmila Ivanova, Elena Solodova, Nadezhda Korotetskaya, Alfia Tugeyeva, Timur Izmailov

**Affiliations:** 1 Department Biochemical Engineering, LLC International Engineering and Technological University, 050060 Almaty, Kazakhstan; 2 JSC Scientific Center for Anti-Infectious Drugs, 050060 Almaty, Kazakhstan; 3 LLP Research and Production Association Kazpharmacom, 040900 Irgeli, Kazakhstan

**Keywords:** resistome, antimicrobial resistance, cloacal microbiota, poultry, pooled metagenomics

## Abstract

Monitoring poultry microbiota and antimicrobial resistance genes is important, as they can reflect flock health, farm conditions, and the level of antimicrobial resistance. Although shotgun metagenomics has been widely applied worldwide to investigate poultry microbiota and antimicrobial resistance, comparable baseline datasets describing the cloacal microbiota and resistome of poultry in Kazakhstan are scarce. In this study, taxonomic and resistome profiles were characterized in pooled metagenomes of the cloacal microbiota of chickens sampled from household and industrial poultry farms in Kazakhstan. Cloacal swabs were collected from laying hens, pooled at the house level, and analyzed using high-throughput metagenomic sequencing. Taxonomic profiles were generated at the genus level, and antimicrobial resistance gene signals were summarized by drug class. Compositional patterns were assessed using CLR/Aitchison ordination, the Mantel test, and Procrustes analysis. The pooled samples exhibited heterogeneous microbiota profiles at the genus level and included taxa of veterinary interest, such as *Chlamydia*, *Avibacterium*, and *Gallibacterium* spp. Resistome profiling revealed a broad but uneven distribution of antimicrobial resistance signals, including those associated with tetracyclines, fluoroquinolones, aminoglycosides, and beta-lactams. Taxonomic and resistome profiles showed preliminary alignment at the matrix level, indicating that resistome variations are partially linked to microbial community structure.

## Simple Summary

Chickens are an important source of meat and eggs, and their health is closely linked to food safety and poultry production efficiency. Microorganisms living in and around chickens can support normal digestion and protect against disease; however, some may also pose health risks or carry genes linked to resistance to antimicrobials used against bacterial infections. In this study, we examined pooled cloacal swab samples from chickens on household and industrial poultry farms in Kazakhstan. We used shotgun metagenomic sequencing to describe the main microbial taxa and identify signals associated with antimicrobial resistance genes. The pooled samples collected from different poultry farms and geographic regions showed heterogeneous microbial community compositions and included several taxa of veterinary interest, such as *Chlamydia*, *Avibacterium*, and *Gallibacterium* spp. We also detected a broad but uneven distribution of resistance-related signals. The results suggest that shotgun metagenomic analysis of pooled cloacal swab samples can help identify poultry houses that may require closer veterinary attention. However, these findings are screening signals and should be confirmed by targeted laboratory tests before being used for diagnosis or treatment decisions.

## 1. Introduction

Poultry farming is an important part of global food systems, providing an affordable source of animal protein and contributing to food security and nutrition [1,2]. Poultry health affects productivity, animal welfare, biosecurity, and food safety. The gastrointestinal tract of chickens contains a complex microbiota that is important for digestion, nutrient metabolism, immune system development, and protection against harmful microorganisms [3,4]. Its composition is influenced by factors such as age, diet, housing conditions, hygiene, and exposure to antimicrobial drugs [5]. Poultry microbiota may include commensal and environmental microorganisms, as well as opportunistic and pathogenic taxa. Genera such as *Chlamydia*, *Avibacterium*, *Gallibacterium*, *Campylobacter*, and *Salmonella* are of veterinary importance because they include species associated with poultry diseases and foodborne transmission [6,7,8,9]. In addition to taxonomy, poultry microbial communities serve as important reservoirs of antimicrobial resistance genes (ARGs). Antimicrobial resistance in poultry develops under the influence of antimicrobial use on poultry farms, microbial composition, environmental factors, and biosecurity measures [10,11,12]. From the perspective of the “One Health” concept, ARG monitoring is important because resistance determinants can circulate among animals, humans, food products, and the environment.

Previous studies conducted in Kazakhstan have described regional variations in the chicken microbiome and provided initial metagenomic data on poultry-associated microbial communities [13,14,15]. Phenotypic antimicrobial resistance has also been reported in *Salmonella enterica* isolates recovered from poultry products in Northern Kazakhstan [16]. However, integrated studies combining cloacal microbiota profiles with the metagenomic characterization of ARG-associated signals across different poultry production systems in Kazakhstan remain limited.

Traditional culture-based methods and PCR continue to play key roles in pathogen confirmation, but they provide limited information about the overall composition of microbial communities. Shotgun metagenomic sequencing allows for simultaneous determination of taxonomic composition and functional characteristics, including the presence of antibiotic resistance genes (ARGs) [12,17]. A comprehensive analysis of taxonomic and resistome profiles helps us better understand the ecology of antimicrobial resistance, as the distribution of antibiotic resistance genes (ARGs) may partially reflect the bacterial communities that carry them. Therefore, comparing taxonomic and resistome structures helps identify community-driven patterns and highlight signals that warrant further investigation.

This exploratory study was motivated by the need to characterize the poultry gut microbiome and antimicrobial resistance (AMR) profiles in Kazakhstan, given the importance of understanding the diversity and distribution of resistance determinants in food-producing animals and their potential implications for animal health, food safety, and public health. The study aimed to characterize the taxonomic composition and resistome profiles of pooled metagenomes from cloacal samples of chickens, and to assess the relationship between microbiota composition and resistome structure. The findings provide baseline information for monitoring the poultry microbiome and AMR in Kazakhstan and support the identification of priority targets for subsequent molecular, culture-based, and epidemiological investigations.

## 2. Materials and Methods

### 2.1. Sample Collection

**Figure 1:**
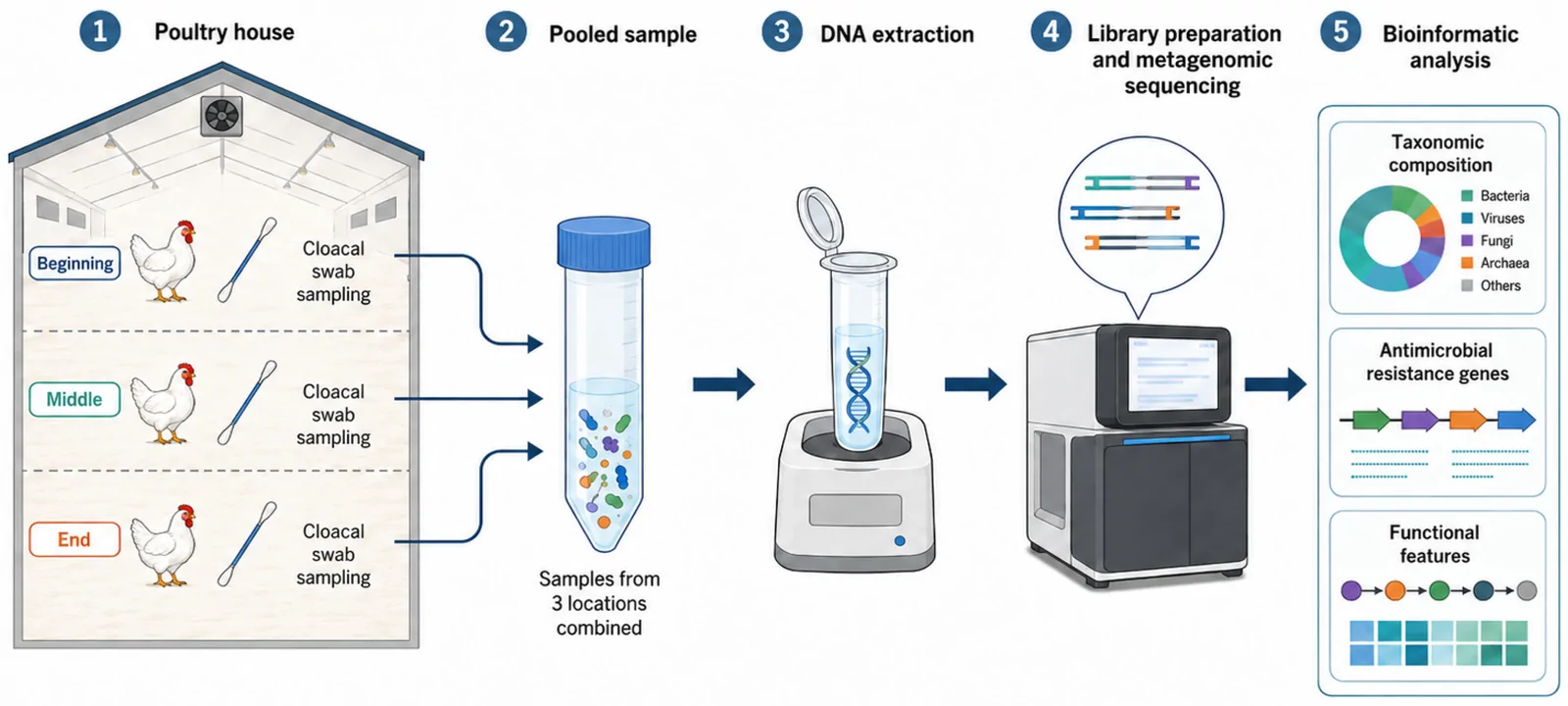
Sampling design and shotgun metagenomic workflow for pooled cloacal swab samples. Nine cloacal swabs collected from three spatial positions within each poultry house were combined into one analytical sample. DNA was extracted from each pool, sequenced using the Ion Torrent PGM platform, and analyzed for genus-level taxonomic composition and ARG-associated read-mapping signals.

All cloacal swab samples included in this study were obtained from laying hens kept in both household and industrial poultry farms. Cloacal swabs were collected using sterile cotton swabs dipped in 2 mL of transport medium (Viral Specimen Collection Tube, Shandong Chengwu Medical Products Factory, Heze, China). Cloacal swabs were collected from hens at three spatially distinct locations within each poultry house: the beginning, middle, and end. At each of the three sampling locations within the same poultry house, samples were collected from three birds per location. The nine swabs were combined to generate a pooled sample. Each pooled sample represented the integrated cloacal microbial profile of the birds from a given poultry house (Figure 1). Fifteen pooled samples were generated and included in this study. Each pooled sample represented one poultry house sampled at a specific location, bird age, and sampling date and was treated as a single analytical unit throughout the study. Samples obtained from the same region but with different metadata (e.g., host age or sampling date) were labeled with a number (Table 1). The samples were transported overnight at 4 °C in Viral Specimen Collection Tubes under refrigerated conditions, according to the manufacturer’s recommendations for short-term sample transport. Upon arrival at the laboratory, all samples were immediately stored at −80 °C until DNA extraction. All samples were processed using the same transport protocol to ensure consistency across the sampling sites. Each pooled sample was considered an independent unit of observation. Farm type (household or industrial), region, host age, breed, and sampling date were recorded as descriptive metadata and were not used to define experimental comparison groups.

### 2.2. Ethics Statement

No experimental procedures were performed on animals. Cloacal swabs were collected as part of field sampling under the approved protocol by the Committee of Institutional Animal Care and Use in the Scientific and Production Center of Microbiology and Virology LLP, Almaty (ID: # 02-09-47 from 27 October 2022).

### 2.3. Sequencing

Total DNA was extracted from samples using the PureLink Genomic DNA Mini Kit (Invitrogen, Carlsbad, CA, USA) following the manufacturer’s protocol. The extracted DNA was subsequently used for library preparation and high-throughput metagenomic sequencing. The quality and quantity of the resulting DNA samples were determined using a NanoDrop 2000c spectrophotometer (Thermo Fisher Scientific, Wilmington, DE, USA) at optical wavelengths of 260 and 280 nm. DNA samples were stored frozen (−20 °C) until use.

The DNA library was sequenced using the Ion Torrent PGM sequencing platform following the manufacturer’s protocol. Briefly, the sequencing library was prepared using the Ion Xpress Plus Fragment Library Kit (Life Technologies, Carlsbad, CA, USA). DNA fragment barcoding for multiplex sequencing was performed using the Xpress Barcode Adapters Kit (Life Technologies, USA). Sequencing of the resulting library was performed on a 314 chip using the Ion PGM Hi-Q View Sequencing Kit. The raw metagenomic data generated in this study are available in the NCBI database under BioProject PRJNA1467164 (https://www.ncbi.nlm.nih.gov/bioproject/PRJNA1467164 accessed on 16 May 2026).

### 2.4. Bioinformatic Analysis

Further processing of the DNA reads was performed using the software tools and parameters described below. Taxonomic classification was performed using Kaiju (version 1.10.2) [18] implemented in KBase (version 5.5.0) [19]. The analysis was run in Greedy mode against the NCBI BLAST nr (updated: 25 August 2024) reference database without eukaryotes. The following parameters were used: low-abundance filtering enabled with a 0.5% threshold, low-complexity filtering enabled, minimum match length = 15, minimum match score = 65, maximum e-value = 0.01, and three allowed mismatches. The resulting genus-level taxonomic classification tables were exported from KBase for downstream analysis in R.

The molecular resistome was characterized using Resistance Gene Identifier bwt (RGI bwt, version 6.0.5). FASTQ reads were mapped using the KMA aligner against CARD protein homolog model reference sequences from the Comprehensive Antibiotic Resistance Database (CARD, version 4.0.1) [20]. The *--include_wildcard* option was used to include predicted allelic variants from the WildCARD Resistomes and Variants dataset (version 4.0.2), thereby expanding the reference space for AMR gene detection in non-clinical metagenomic samples. RGI bwt gene-level output was exported for downstream analysis. The complete gene-level output for all pooled samples, including ARO terms and accessions, mapped read counts, reference protein identity, average sequence coverage, AMR gene family, drug class, and resistance mechanism, is provided in Supplementary Dataset S1. No additional read count, MAPQ, or reference coverage thresholds were applied before summarizing the drug class level. Therefore, individual entries in Supplementary Dataset S1 were interpreted as ARG-associated read-mapping signals rather than confirmed complete or functionally active resistance genes. All downstream analyses were performed in R version 4.5.3 [21] using the packages *readxl* [22], *tidyverse* [23], *janitor* [24], *vegan* [25], *ggrepel* [26], *ComplexUpset* [27], *scales* [28], *RColorBrewer* [29], and *writexl* [30]. Input files included a taxonomic abundance matrix and an antimicrobial resistance matrix.

To visualize the regional taxonomic landscape, taxon abundance was converted to relative abundance by dividing each taxon count by the total count per sample. The 30 most abundant genera were selected according to the mean relative abundance across all samples, while all remaining taxa were collapsed into a single category labeled “Other”. A stacked bar plot was generated to display the relative genus composition of each pooled sample.

For each pooled sample and drug class, the total number of mapped reads was calculated as the sum of *all_mapped_reads*, mean coverage was calculated from *average_percent_coverage*, and the number of distinct ARGs per class was estimated from *aro_term* when available, or from *drug_class*. This summarized table was used to construct a drug class by a pooled sample matrix of total mapped reads.

Resistome signatures were visualized as a heatmap based on the drug class matrix. Drug classes were ordered by decreasing total read abundance across all samples, and heatmap fill values were calculated as log10 reads.

Because sequencing-derived taxonomic and resistome profiles are compositional, ordination analyses were performed in the CLR-transformed space. Prior to transformation, a pseudocount of 0.5 was added to all entries in both the taxonomic and drug class matrices to avoid undefined logarithms for zero-value entries. CLR transformation was implemented by log-transforming the pseudocount-corrected matrix and centering each sample by its mean log abundance. The Euclidean distances between the CLR-transformed sample profiles were then calculated and interpreted as Aitchison distances. Principal coordinates analysis (PCoA) was performed using *cmdscale*() with the extraction of the first two axes, and the percentage of explained variation was calculated from positive eigenvalues only. This strategy follows current recommendations for the analysis of compositional microbiota and metagenomic data [31].

To interpret the PCoA results, we examined the features associated with ordination axes. For taxonomic PCoA, CLR-transformed genus abundances were correlated with the sample scores on PCoA1 and PCoA2. For the resistome PCoA, CLR-transformed AMR drug class abundance was correlated with the corresponding PCoA axes. Pearson’s correlation coefficients were used to identify the features that were most strongly associated with each axis. Because only one pooled sample was available per region and no biological replicates were included, these correlations were interpreted descriptively as ordination axis associations rather than as evidence of statistically significant effects.

To assess how closely the microbial community composition corresponded to the structure of the resistome, only samples present in both taxonomic and drug class datasets were included in the analysis. The relationship between taxonomic composition and resistome profiles was first evaluated using a Mantel test implemented in the vegan package (*mantel*) with Spearman’s correlation and 9999 permutations. This test was used to determine whether samples that differed more in their microbial composition also differed more in their resistome composition. To compare the overall patterns across the two datasets, principal coordinate analysis (PCoA) was performed separately on taxonomic and drug-class distance matrices. The resulting ordinations were compared using symmetric Procrustes analysis, which aligns the two configurations and measures their similarity. The statistical significance of this similarity was assessed using PROTEST (*protest*) with 9999 permutations.

## 3. Results

### 3.1. Sample Overview

**Figure 2:**
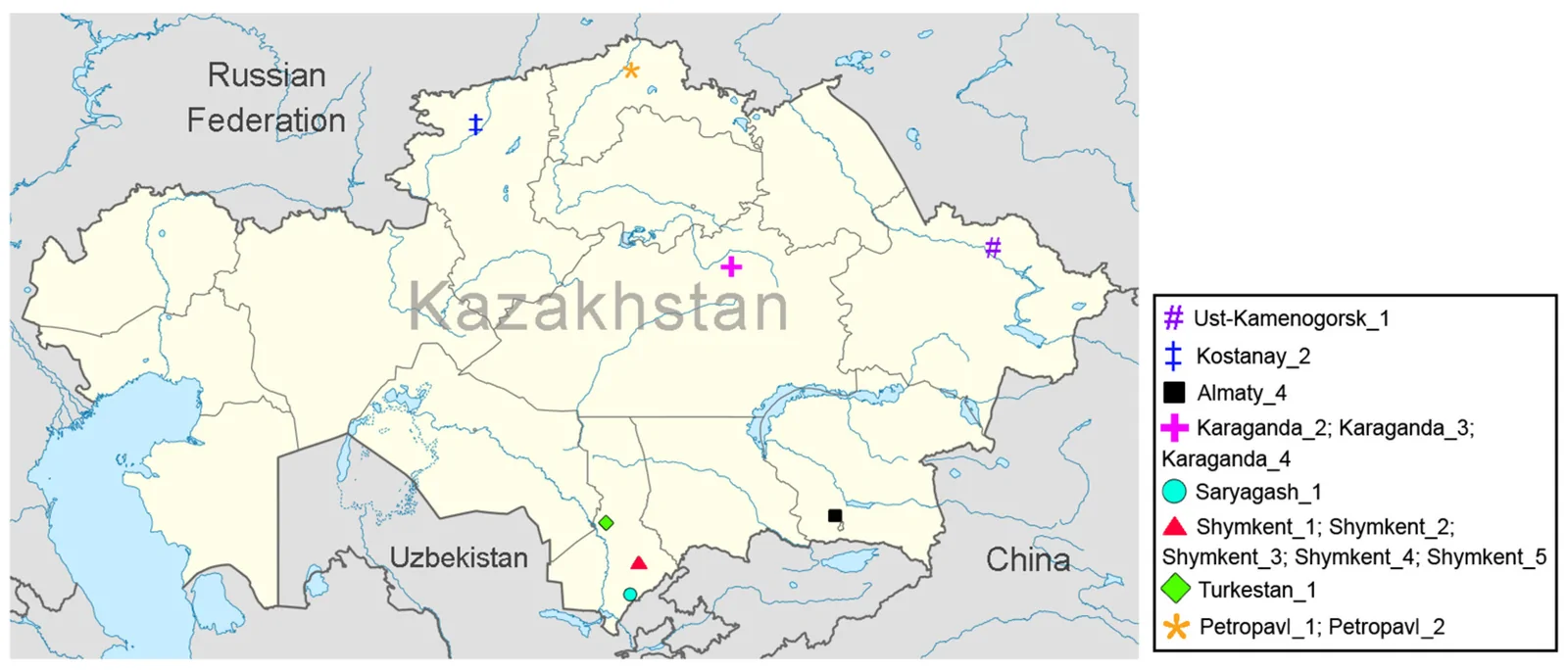
Geographic distribution of poultry sampling sites across Kazakhstan. The map shows the locations of the poultry farms from which the 15 pooled cloacal samples were obtained. Sampling sites were distributed across eight regions of Kazakhstan, and colors and symbols identify individual sampling locations.

Fifteen pooled cloacal metagenomes representing household and industrial poultry farms from eight regions of Kazakhstan were included in the analysis. Sampling sites were distributed across eight regions of Kazakhstan (Figure 2), while detailed farm and bird metadata are provided in Table 1.

Because the sampling sites differed not only by geographic region but also by the age of the birds, the date of sampling, cross, and type of farm, the observed differences should be interpreted at the level of the samples or in relation to specific farms, rather than as isolated geographic effects.

### 3.2. Sequencing

Sequencing generated 35,651–362,444 single-end reads per sample, with mean read lengths ranging from 128.91 to 181.19 bp and mean quality scores ranging from 27.46 to 31.17 (Table 2).

The sequencing depth varied substantially among the samples, ranging from 35,651 to 362,444 reads per sample. Therefore, downstream comparisons were interpreted primarily as exploratory compositional patterns rather than quantitative estimates of absolute microbial or ARG loads. Based on this constraint, we first examined the dominant genus-level taxonomic profiles of the pooled samples.

### 3.3. Taxonomic Profiles

**Figure 3:**
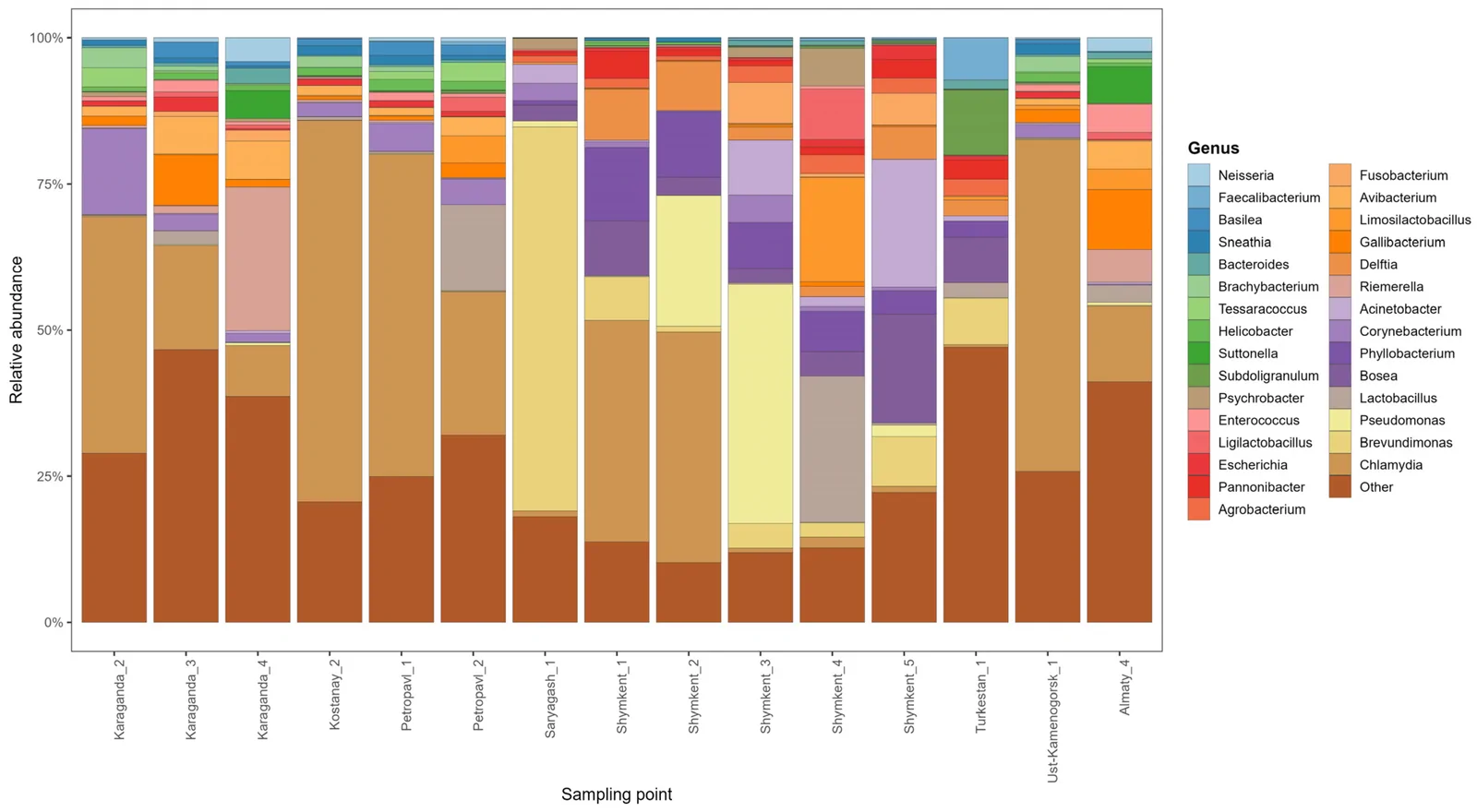
Genus-level taxonomic composition of pooled cloacal metagenomes from poultry farms in Kazakhstan. Each bar represents one pooled sample, and colors indicate the relative abundance of the 30 most abundant genera; all remaining genera are combined as “Other”.

When compiling the taxonomic profile, a filter with a threshold of 0.5% was applied. Therefore, the analysis primarily reflected dominant and moderately widespread taxa rather than the entire rare biosphere as a whole (Figure 3).

The relative contribution of the 30 most abundant genera differed markedly among the pooled samples, accounting for 52.9–89.8% of the genus-level taxonomic profiles. These differences indicate substantial heterogeneity in microbial community composition among the analyzed samples (Figure 3).

Among the detected genera, several taxa were of particular veterinary interest because they included species associated with poultry respiratory or reproductive disorders. *Chlamydia*-associated reads were detected in all 15 pooled samples, but their relative abundance varied markedly among the pooled samples. The genus accounted for between 8 and 1691 assigned reads per sample, corresponding to 0.44–65.29% of the taxonomic profile at the genus level. The highest *Chlamydia* signal was observed in Kostanay_2 (1691 reads, 65.29%), followed by Ust-Kamenogorsk_1 (1192 reads, 56.79%), and Petropavl_1 (1335 reads, 55.14%).

Among the poultry-associated respiratory genera, *Avibacterium* was detected in 10 of 15 samples, with a total of 359 assigned reads. In positive samples, read counts ranged from 2 to 81 reads, corresponding to relative abundances of 0.07–6.52%. The highest relative abundance was observed in Karaganda_4 (69 reads, 6.52%), whereas the highest read count was observed in Petropavl_2 (81 reads, 3.23%).

*Gallibacterium* was detected in 13 of the 15 samples, with 353 reads assigned to it. In positive samples, the number of reads ranged from 4 to 62, corresponding to a relative abundance of 0.15–10.25%. The strongest relative signal was observed in Almaty_4 (62 reads, 10.25%), followed by Karaganda_3 (61 reads, 8.73%).

These taxonomic patterns provide a microbial community context for interpreting antimicrobial resistance signals. Therefore, we next examined ARG-derived resistome profiles at the antimicrobial drug-class level.

### 3.4. Resistome Profiles

**Figure 4:**
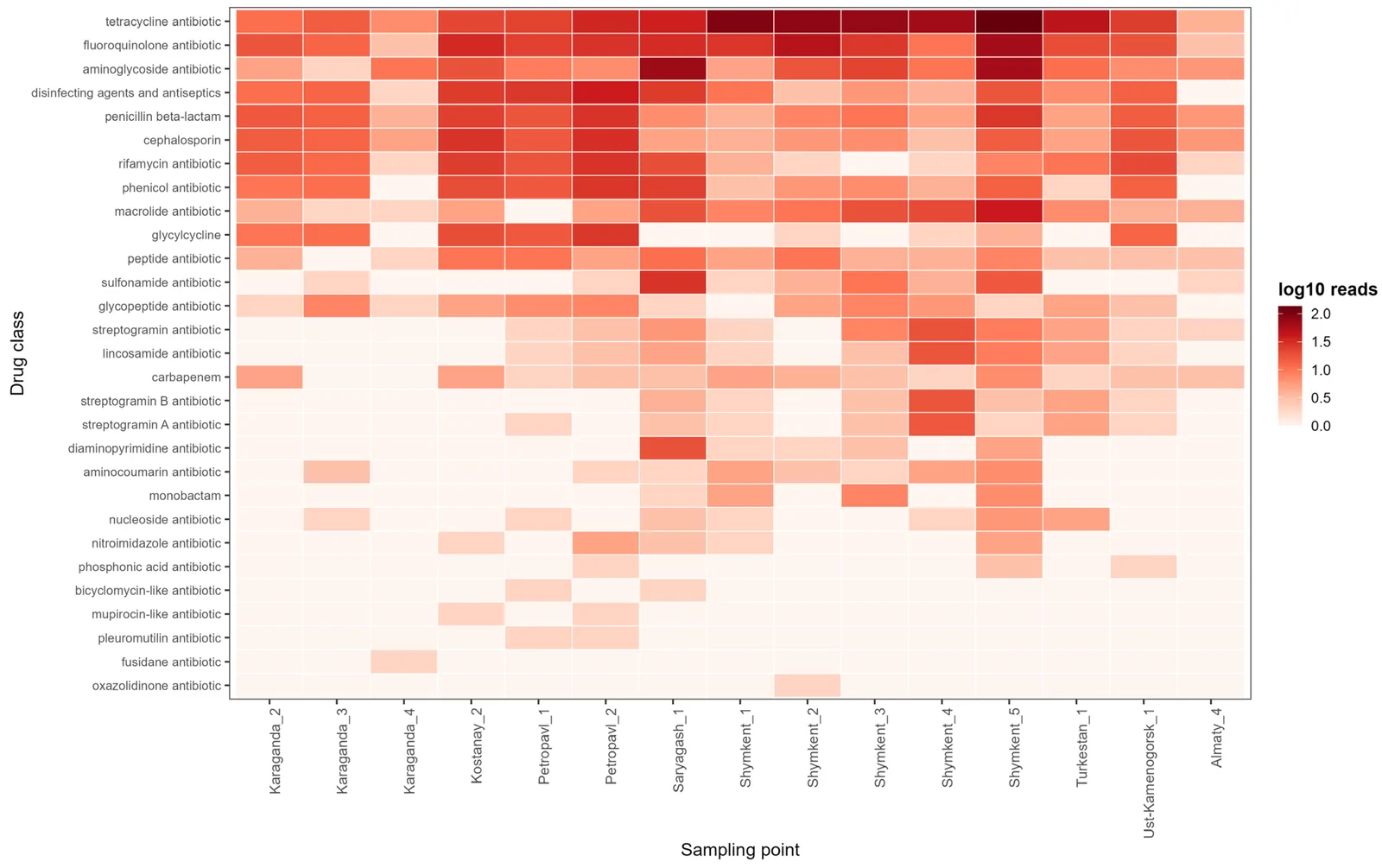
Distribution of ARG-associated read-mapping signals across antimicrobial drug classes. Columns represent the 15 analytical pooled samples, each generated by combining nine cloacal swabs collected from three within-house collection locations in one poultry house. Rows represent antimicrobial drug classes. Color intensity corresponds to the log10-transformed number of mapped reads, with darker cells indicating stronger ARG-associated signals.

In addition to taxonomic profiling, antimicrobial resistance genes were analyzed to characterize the resistome of the pooled poultry samples (Figure 4). Particular attention was given to drug classes of veterinary and One Health relevance, including tetracycline, fluoroquinolone, aminoglycoside, and beta-lactam resistance genes.

The drug class heatmap revealed a broad but unevenly distributed resistome across the pooled poultry samples. ARG mapping signals associated with 29 antimicrobial drug classes were detected. The gene-level annotations underlying these drug-class summaries are provided in Supplementary Dataset S1. ARG-associated mappings were most frequently assigned to tetracycline, fluoroquinolone, and aminoglycoside resistance classes, whereas the remaining drug classes showed lower and more variable representation among samples (Figure 4).

At the sample level, Shymkent_5 showed the highest overall AMR class read burden and broadest resistance class spectrum, followed by Saryagash_1 and Petropavl_2 samples. Shymkent samples showed a common enrichment of tetracycline-associated resistance signals but differed in additional resistance classes, indicating marked within-region heterogeneity in the resistome structure. Saryagash_1 was characterized by a strong aminoglycoside signal, whereas Petropavl_2 showed pronounced contributions from disinfecting agents and antiseptics, β-lactam-related classes, rifamycin, and phenicol antibiotics.

### 3.5. Compositional Structure

**Figure 5:**
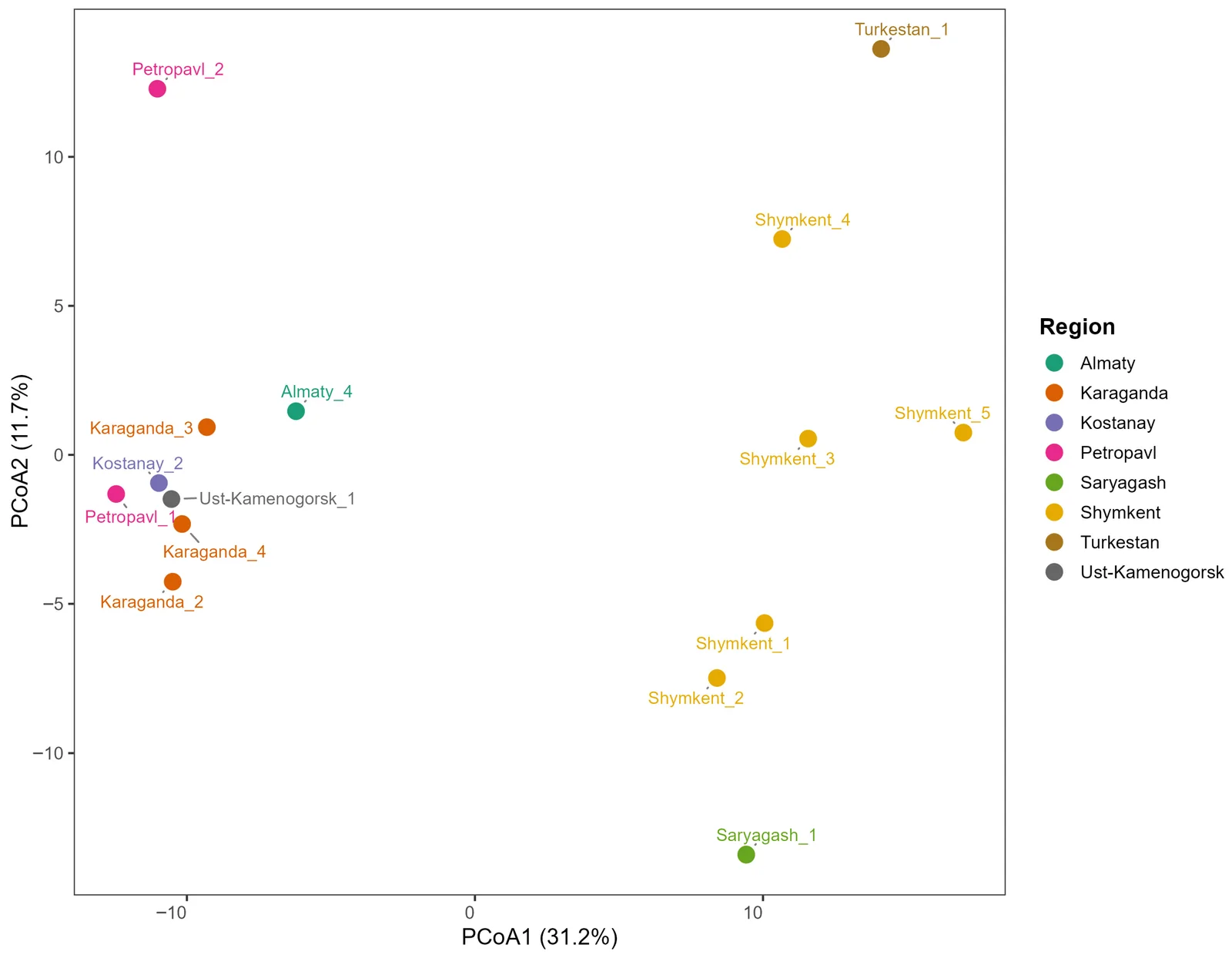
Principal coordinate analysis of genus-level microbial community profiles based on CLR-transformed abundances and Aitchison distances. Each point represents one pooled cloacal sample, and colors indicate the geographic origin of the sample.

**Figure 6:**
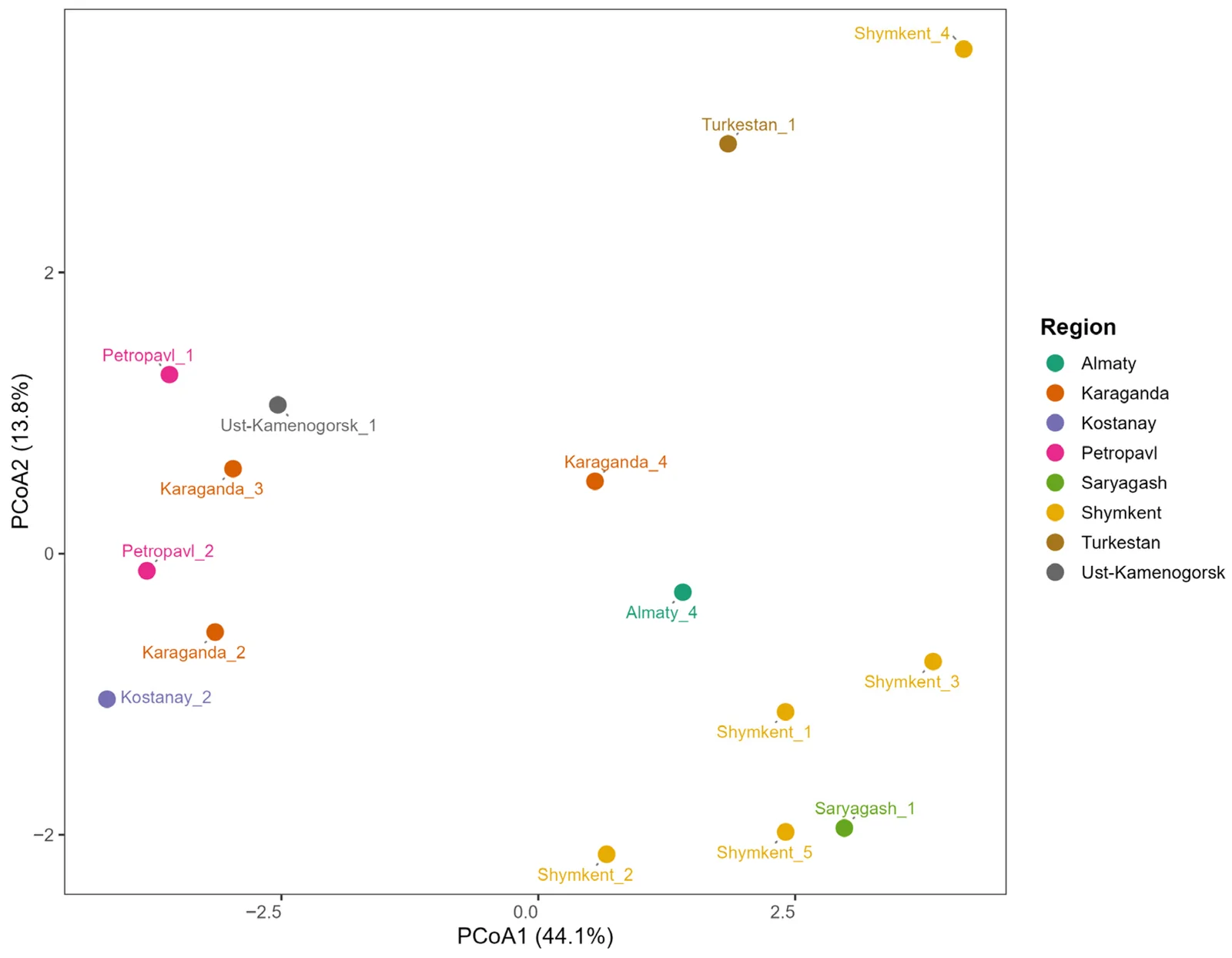
Principal coordinate analysis of drug-class resistome profiles based on CLR-transformed ARG-associated read counts and Aitchison distances. Each point represents one pooled cloacal sample, and colors indicate its geographic origin.

Taxonomic ordination was used to explore differences in microbial community structure among pooled samples (Figure 5), whereas drug-class ordination was used to evaluate class-level resistome signatures (Figure 6).

Principal coordinate analysis based on Aitchison distances of CLR-transformed taxonomic profiles showed exploratory separation among pooled poultry microbiota samples. The first two axes explained 42.9% of the total variation. Samples from Shymkent, Saryagash, and Turkestan were predominantly located on the right side of PCoA1, whereas samples from Karaganda, Kostanay, Petropavl, Ust-Kamenogorsk, and Almaty were located mainly on the left. This pattern represents sample-level separation rather than a formal geographical test. In addition, the Shymkent samples showed considerable dispersion along the PCoA2, indicating within-region heterogeneity.

After assessing taxon differences, we applied the same methodology to AMR profiles to determine whether resistome composition shows a comparable pattern.

Principal coordinate analysis (PCoA) based on Aitchison distances of CLR-transformed resistome profiles (Figure 6) revealed an exploratory separation pattern among the pooled poultry samples according to their composition of ARG-associated drug-class profiles.

The first two PCoA axes explained 57.9% of the total variation. Samples from Shymkent, Saryagash, and Turkestan predominantly appeared on the positive side of PCoA1, whereas Petropavl, Kostanay, Ust-Kamenogorsk, and most Karaganda samples appeared on the negative side. This pattern indicates differences in the overall drug-class resistome structure of the pooled samples.

Since the ordination of taxon profiles and the resistome revealed a clustering of individual samples into distinct groups, we assessed the relationship between these two configurations.

**Figure 7:**
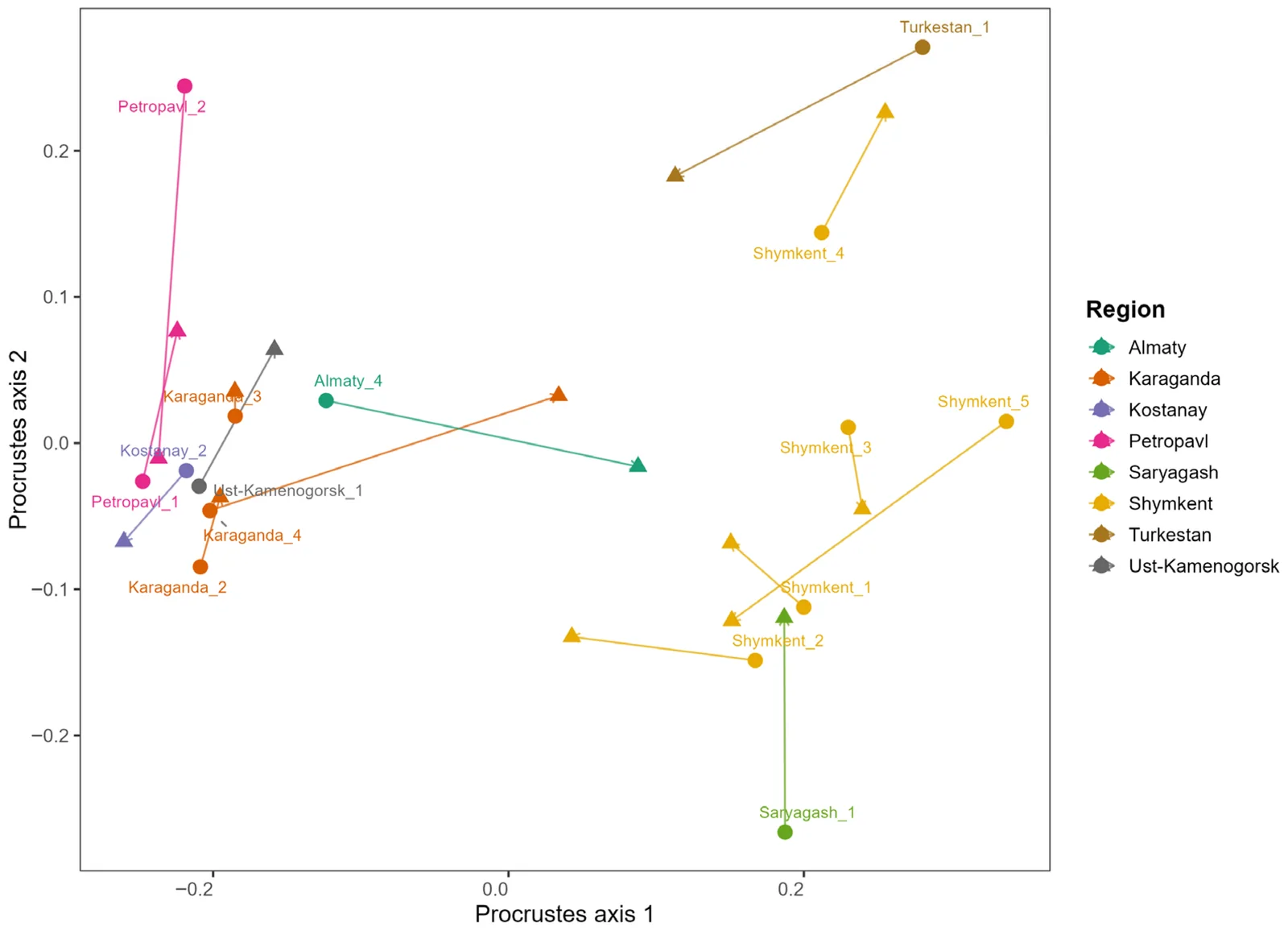
Procrustes comparison of taxonomic and drug-class resistome ordinations in pooled cloacal metagenomes. Circles represent sample positions in the taxonomic ordination, and triangles represent the corresponding resistome ordination after Procrustes rotation. Arrows connect paired positions for the same, pooled sample. Longer arrows indicate greater discordance between the taxonomic composition and drug class resistome structure.

Procrustes analysis was performed to evaluate the concordance between the CLR/Aitchison ordinations of the taxonomic and resistome profiles (Figure 7).

An exploratory matrix-level concordance was observed between the taxonomic and drug class resistome profiles (PROTEST r = 0.809, *p* = 2 × 10^−4^). Although permutation-based Mantel and PROTEST analyses suggested concordance between the two data structures, these results should not be interpreted as evidence of causality or formal regional effects, as each poultry house was represented by a single pooled metagenome (Table 3). These results indicate that pairwise differences in taxonomic composition were positively associated with pairwise differences in drug-class resistome composition.

These findings indicate matrix-level concordance between the taxonomic and drug-class resistome profiles, with residual differences suggesting an incomplete overlap between the two compositional structures.

## 4. Discussion

In this study, taxonomic profiles and antimicrobial resistance gene (ARG) signals were jointly characterized in pooled cloacal samples collected from chickens in different regions of Kazakhstan. The microbial community composition varied markedly among the pooled samples. Several genera of potential veterinary relevance were also detected, including *Chlamydia*, *Avibacterium*, and *Gallibacterium*. ARG-associated signals covered a wide range of antimicrobial classes but were unevenly distributed across the samples. Tetracycline-associated sequences were detected most frequently. In addition, differences in microbiota composition were associated with differences in the resistome structure.

These findings should be interpreted as characteristics of individual pooled samples rather than evidence of regional differences. The pooled samples differed not only in geographic location but also in bird age, breed, farm type, sampling date, and sequencing depth. Moreover, independent biological replicates were unavailable for the regions included in this study. Consequently, the effects of geographic location cannot be separated from those of other factors.

The pronounced taxonomic variability observed among the samples was consistent with previous studies. The chicken intestinal microbiota is influenced by age, genetic background, diet, housing conditions, antimicrobial exposure, and contact with the farm environment [3,4,5,6,12,13,32]. *Firmicutes*, *Bacteroidota*, *Proteobacteria*, and *Actinobacteriota* are commonly reported as major components of the chicken intestinal microbiota. However, their relative proportions and dominant genera can vary substantially among breeds, geographic regions, production systems, and sections of the gastrointestinal tract [13,32,33,34]. For example, Xu et al. identified breed-associated differences in both microbiota composition and ARG profiles in Chinese chickens [12]. Analyses of large metagenomic collections have revealed considerable heterogeneity among individual samples and geographic regions [33,34]. Therefore, the separation observed in the ordination analysis was biologically plausible. Nevertheless, this cannot be attributed solely to geography because the region was closely associated with bird age, breed, farm type, and sampling date in the present study.

One of the most notable findings was the detection of sequences assigned to the genus *Chlamydia* in all the pooled samples. The highest relative abundances were recorded for Kostanay_2, Ust-Kamenogorsk_1, and Petropavl_1. The genus *Chlamydia* includes species of veterinary and zoonotic significance [7]. However, the genus-level classification of short metagenomic reads does not allow the identification of a specific species. Moreover, it does not confirm the presence of viable organisms, active infection, or clinical disease. Therefore, the detected sequences should be regarded as signals requiring further investigation rather than confirmation of chlamydial infection. More precise taxonomic identification would require species-specific quantitative PCR, sequencing of diagnostically informative genomic regions, and culture-based or serological testing, where appropriate material is available.

The detection of *Avibacterium* and *Gallibacterium* also requires careful interpretation. *Avibacterium paragallinarum* is primarily associated with the upper respiratory tract and is the causative agent of infectious coryza in chickens. In contrast, *Gallibacterium anatis* may occur in the respiratory and reproductive tracts as a commensal or opportunistic pathogen [8,9,35,36,37]. The detection of these genera in cloacal samples does not indicate respiratory or reproductive diseases. Possible explanations include the passage of microorganisms or their DNA through the gastrointestinal tract, contact of the cloaca with litter and other environmental materials, or contamination with respiratory or reproductive secretions. Therefore, the clinical relevance of these findings can only be assessed by examining samples collected directly from the relevant anatomical sites using species-specific PCR and culture-based methods.

Close contact between birds and the poultry house environment must also be considered when interpreting cloacal metagenomes. Chickens are continuously exposed to litter, feces, dust, feed, and water. Consequently, cloacal swabs may contain not only stable members of the intestinal microbiota but also transient microorganisms originating from the environment. Studies comparing the microbiota of the intestine, feces, and litter have shown that these communities differ in composition, although microorganisms are continuously exchanged among them [38,39,40,41]. The litter microbiota also changes throughout the production cycle and is influenced by moisture, pH, ammonia concentration, stocking density, litter reuse, and flock management practices. Environmental or litter-associated taxa detected in the present study should, therefore, not be automatically interpreted as laboratory contamination. However, their presence does not demonstrate stable intestinal colonization. A pooled cloacal sample is more likely to reflect the combined microbial backgrounds of birds and their environment. This approach may be useful for farm-level screening, although it has limited ability to identify the precise source of each microorganism.

Sequences associated with ARGs corresponding to 29 antimicrobial classes were detected in the samples examined. Tetracycline-associated ARGs accounted for the largest number of mapped reads, followed by fluoroquinolone- and aminoglycoside-associated signals. A similar resistome structure has been reported in other poultry studies. Xu et al. showed that ARGs associated with tetracyclines, aminoglycosides, and multidrug resistance represented a substantial proportion of the resistome in Chinese chickens [12]. Yang et al. identified genes associated with resistance to tetracyclines, aminoglycosides, and macrolides as the predominant ARG categories in an analysis of 629 chicken gut metagenomes [33]. Tetracycline- and macrolide-associated ARGs were also among the most common categories in metagenomic collections of chicken samples from China and several European countries [34]. Thus, the high representation of tetracycline-associated ARGs in our samples is consistent with patterns previously reported for the poultry intestinal resistome and does not appear to be unique to Kazakhstan.

The widespread occurrence of tetracycline-associated ARGs may have several explanations. Tetracyclines have been used in livestock production for many years in numerous countries, including Kazakhstan, and resistance genes selected during previous antimicrobial exposure may persist in microbial communities even after the use of these drugs is reduced or discontinued. Tetracycline-class antimicrobials remain in veterinary use in Kazakhstan, with a recent national survey identifying oxytetracycline among the antibiotics most frequently dispensed by veterinary pharmacies [42]. In addition, many tetracycline resistance genes are associated with plasmids, transposons, and other mobile genetic elements. These elements facilitate the transfer between bacterial hosts and may promote long-term persistence within microbial communities [33,34]. The farm environment, particularly feces and reused litter, may provide additional routes for the introduction and maintenance of ARGs in poultry populations [38,39,40,41].

However, data on antimicrobial use on the farms included in this study were unavailable. Therefore, it was not possible to determine whether the detected tetracycline-associated ARGs were related to the recent use of these drugs. It cannot be concluded that tetracyclines are currently the most frequently used antimicrobial class in Kazakhstan. ARG signals associated with fluoroquinolones and aminoglycosides were also detected in all pooled samples, whereas sequences associated with β-lactams, phenicols, rifamycins, macrolides, and disinfecting agents showed a less uniform distribution.

Experimental studies have demonstrated that antimicrobial administration may alter both intestinal microbiota and the resistome. Nevertheless, ARGs and phenotypically resistant bacteria may persist in litter and cloacal samples even when the corresponding antimicrobials are not administered during the current production cycle [38,43]. Therefore, the resistome profiles observed in this study may reflect a combination of recent and historical antimicrobial exposure, introduction of microorganisms through birds and environmental sources, persistence of ARGs in litter and poultry house environments, and the original composition of the microbial community.

The highest number of ARG-associated reads was recorded for Shymkent_5. However, the samples collected from Shymkent differed markedly in the additional ARG classes they contained. This within-region variation suggests that differences among individual flocks, poultry houses, or sampling periods may be at least as important as the geographic location of the farm.

The Mantel test and Procrustes analysis indicated a concordance between taxonomic composition and resistome structure. In other words, samples that differed more strongly in microbial composition generally also differed more strongly in the distribution of ARGs among the antimicrobial classes. Similar relationships have been reported in large poultry metagenomic datasets [33,34]. This association is biologically plausible because different bacterial groups carry distinct sets of intrinsic and acquired resistance genes.

Nevertheless, taxonomic composition is not the only factor that shapes the resistome. Yang et al. identified members of the genera *Escherichia*, *Enterococcus*, *Staphylococcus*, and *Klebsiella*, as well as several lactic acid bacterial genera, as potential ARG hosts in the chicken intestine [33]. They also reported an association between ARG abundance and the abundance of mobile genetic elements. Feng et al. found that plasmid composition explained resistome variation more effectively than overall taxonomic composition in some datasets [34]. Therefore, the incomplete agreement between taxonomic and resistome ordinations may reflect differences in plasmids and other mobile elements, strain-specific ARG content, horizontal gene transfer, and the introduction of ARGs from environmental sources. These processes cannot be fully resolved using genus-level taxonomic classification or drug-class-level ARG summaries alone.

An important limitation of the present study is that metagenomic analysis detected sequences similar to known ARGs but did not determine the phenotypic antimicrobial resistance of the bacterial isolates. The detection of an ARG-associated sequence does not demonstrate that the corresponding gene is complete, functionally active, or expressed. It also cannot establish whether the sequence is present in a viable bacterium or whether it confers phenotypic resistance to an antimicrobial agent.

This study has several limitations. Nine cloacal swabs were combined to form a single analytical sample. This approach provided an integrated description of each poultry house and reduced the influence of individual birds on the results. However, it does not allow the assessment of individual variation or the prevalence of particular taxa or ARGs within a flock. Independent biological replicates were not available for all regions and farm categories. Therefore, formal comparisons between regions or between household and industrial poultry farms were not possible.

The sequencing depth also varied substantially among the samples. Transport at 4 °C rather than on dry ice may have altered the relative abundance of some low-abundance or temperature-sensitive microorganisms, although the standardized overnight transport protocol likely reduced the systematic bias among samples. In addition, the relatively short single-end reads limited the reconstruction of complete genes and mobile genetic elements and prevented the reliable assignment of ARGs to specific bacterial hosts. Interpretation was further restricted by the absence of standardized metadata on antimicrobial use, clinical condition of the birds, vaccination, diet, litter characteristics, and biosecurity practices. Therefore, the findings should be regarded as baseline information for epidemiological and veterinary surveillance rather than quantitative estimates of regional ARG prevalence or absolute antimicrobial resistance burden.

Despite these limitations, this study expands the available knowledge of the microbiota and resistome of chickens from household and industrial poultry farms in Kazakhstan, where comparable metagenomic data are limited. A joint analysis of taxonomic composition and ARG-associated sequences can help to identify pooled samples and their corresponding poultry houses that warrant more detailed veterinary investigations.

Future metagenomic surveillance should include repeated sampling, independent biological replicates, standardized recording of bird age and production systems, deeper sequencing, and parallel analysis of litter, water, feed, dust, respiratory tract, and reproductive tract samples. The additional use of quantitative PCR, culture-based methods, phenotypic antimicrobial susceptibility testing, metagenomic assembly, and mobile genetic element analysis would help clarify the origin of the detected signals and provide a more accurate assessment of their veterinary and epidemiological significance.

## 5. Conclusions

This study provides an integrated shotgun metagenomic assessment of cloacal microbiota and ARG-associated signals in chickens from household and industrial poultry farms in Kazakhstan. The pooled samples showed heterogeneous microbial communities, contained taxa of veterinary relevance, and differed in resistome composition. However, the absence of independent biological replication prevents estimation of prevalence, causal inference, and robust comparisons among regions or farm types. Therefore, the detected signals should be regarded as screening findings requiring confirmation by targeted molecular assays, culture, phenotypic antimicrobial susceptibility testing, or deeper sequencing. Although shotgun metagenomics should not yet be considered a stand-alone diagnostic method, it may complement routine surveillance by revealing unexpected microbial or ARG-associated signals and prioritizing samples for targeted investigations. As sequencing costs decline and analytical workflows become more standardized, the feasibility of periodic sentinel surveillance may increase. These findings provide a baseline for future monitoring of the poultry microbiome and resistome in Kazakhstan.

## Figures and Tables

**Table 1:** Summary table of farm and bird characteristics in the regions of Kazakhstan.

#	Region	Sampling Date	Farm Type	Host Age	Lat.—Lon.	Cross
1	Almaty_4	14 September 2025	household farms	287 days	43.060 N 76.150 E	arbor acres
2	Karaganda_2	17 February 2024	household farms	334 days	49.513 N 73.153 E	n/d ^1^
3	Karaganda_3	28 October 2024	household farms	585 days	49.513 N 73.153 E	n/d
4	Karaganda_4	3 April 2025	household farms	730 days	49.513 N 73.153 E	n/d
5	Petropavl_1	4 March 2024	household farms	358 days	54.301 N 69.061 E	n/d
6	Petropavl_2	14 October 2024	household farms	578 days	54.301 N 69.061 E	n/d
7	Shymkent_1	15 March 2024	industrial farms	623 days	42.202 N 69.295 E	nick
8	Shymkent_2	15 March 2024	industrial farms	197 days	42.202 N 69.295 E	nick
9	Shymkent_3	15 March 2024	industrial farms	233 days	42.202 N 69.295 E	nick
10	Shymkent_4	15 March 2024	industrial farms	308 days	42.202 N 69.295 E	nick
11	Shymkent_5	15 March 2024	industrial farms	490 days	42.202 N 69.295 E	nick
12	Turkestan_1	15 March 2024	industrial farms	300 days	43.181 N 68.160 E	brown nick
13	Saryagash_1	27 February 2024	industrial farms	196 days	41.275 N 69.105 E	coral
14	Ust-Kamenogorsk_1	14 March 2024	household farms	361 days	49.581 N 82.362 E	n/d
15	Kostanay_2	23 March 2024	household farms	370 days	53.134 N 63.382 E	n/d

^1^ n/d—not determined.

**Table 2:** Sample characteristics.

Region	Number of Reads	Total Number of Bases	Mean Read Length	Quality Score Mean	SRA Number
Almaty_4	35,651	6,152,799	172.58	30.70	SRR38666983
Karaganda_2	168,008	22,597,994	134.50	28.83	SRR38666982
Karaganda_3	74,741	12,472,274	166.87	30.00	SRR38666976
Karaganda_4	65,925	11,945,161	181.19	29.91	SRR38666975
Petropavl_1	362,444	51,693,560	142.62	28.65	SRR38666974
Petropavl_2	300,666	43,709,256	145.37	28.82	SRR38666973
Shymkent_1	161,904	21,636,144	133.63	27.51	SRR38666972
Shymkent_2	125,406	16,165,889	128.91	27.73	SRR38666971
Shymkent_3	58,828	10,630,483	180.70	31.17	SRR38666970
Shymkent_4	55,082	9,298,627	168.81	30.51	SRR38666969
Shymkent_5	111,175	19,449,478	174.94	30.86	SRR38666981
Turkestan_1	49,995	7,556,412	151.14	27.46	SRR38666980
Saryagash_1	60,706	9,107,637	150.02	27.75	SRR38666979
Ust-Kamenogorsk_1	328,165	44,901,533	136.82	29.27	SRR38666978
Kostanay_2	339,241	46,782,229	137.90	28.83	SRR38666977

**Table 3:** Statistical comparison of taxonomic and resistome profiles among pooled samples.

Analysis	Distance	Statistic	*p*-Value	Interpretation
Mantel test	Taxa Aitchison vs. Drug Class Aitchison	r = 0.576	*p* = 0.0004	Taxonomic differences are associated with quantitative differences in the resistome
PROTEST	Taxa PCoA vs. Drug Class PCoA	r = 0.809	*p* = 0.0002	Exploratory permutation analysis indicated concordance between taxonomic and drug-class ordination structures.
Procrustes	Taxa PCoA vs. Drug Class PCoA	RSS = 0.346	—	Residual discrepancy between the two ordination spaces

## Data Availability

The original data presented in the study are openly available in NCBI database at PRJNA1467164.

## Supplementary Materials

The following supporting information can be downloaded at: https://www.mdpi.com/article/10.3390/microorganisms14081689/s1, Supplementary Dataset S1: Gene-level RGI bwt output for ARG-associated read-mapping signals detected in the pooled cloacal metagenomes.

## Abbreviations

The following abbreviations are used in this manuscript:

AMR	Antimicrobial resistance
ARG	Antimicrobial resistance gene
CARD	Comprehensive Antibiotic Resistance Database
CLR	Centered log-ratio
KMA	K-mer alignment
PCoA	Principal coordinate analysis
RGI	Resistance Gene Identifier
SRA	Sequence Read Archive
PROTEST	Procrustes randomization test
